# Drugs acting on the pregnant uterus

**DOI:** 10.1016/j.ogrm.2010.05.001

**Published:** 2010-08

**Authors:** Sarah Arrowsmith, Annabelle Kendrick, Susan Wray

**Affiliations:** **Sarah Arrowsmith BSc MRes** is a PhD Student in the Physiological Laboratory at University of Liverpool, Liverpool, UK. Conflicts of interest: none declared; **Annabelle Kendrick BSc MBChB** is a Specialist Registrar at Liverpool Women’s Foundation Trust Hospital, Liverpool, UK. Conflicts of interest: none declared; **Susan Wray BSc PhD** is a Professor at Liverpool Women’s Foundation Trust Hospital, Liverpool, UK. Conflicts of interest: none declared

**Keywords:** contraction, smooth muscle, tocolytics, uterotonins

## Abstract

In this review we overview the mechanisms responsible for uterine contractility and relaxation. We then use this as the basis for discussing the two major uterotonins, oxytocin and prostaglandins followed by currently available (although often unlicensed) tocolytics; progesterone, magnesium, calcium channel blockers, oxytocin receptor blockers, β-adrenergic receptor agonists, prostaglandin synthesis inhibitors and nitric oxide donors. In this brief review we have concentrated on the most important mechanisms of action and data obtained on human tissue. By focussing on mechanisms, meta-analyses and Cochrane literature reviews, our aim is to provide insight for clinical usefulness, and highlight where further research is required and where the targets may be.

## Introduction

Pregnancy and parturition are natural events but, as with most bodily functions, things can go wrong and nature requires a helping hand. Along with surgical intervention, drugs are the main mechanism used to help control the pregnant uterus. They are used to stimulate the uterus (uterotonins) in three main clinical scenarios: (1) to initiate uterine activity for induction of labour or termination, (2) to augment slowly progressing labours and (3) to stimulate delivery of the placenta and prevent post-partum haemorrhage. They are used to relax the uterus (tocolytics) in cases of threatened or frank preterm labour, to prevent or delay preterm delivery. Due to its frequency and serious adverse effects on neonatal mortality and morbidity, it is preterm labour that is most intensely investigated and for which most new obstetrical drugs are trialled, and which therefore we have spent longer reviewing. An understanding of the mechanisms of action of tocolytics and uterotonins, is therefore essential to aid the management of difficult pregnancies and prevent their associated morbidities. Both classes of drugs target the pathways that initiate and produce uterine contractions. We therefore start with an overview of the basics of these events.

## Uterine excitability and contractility

**Figure 1:**
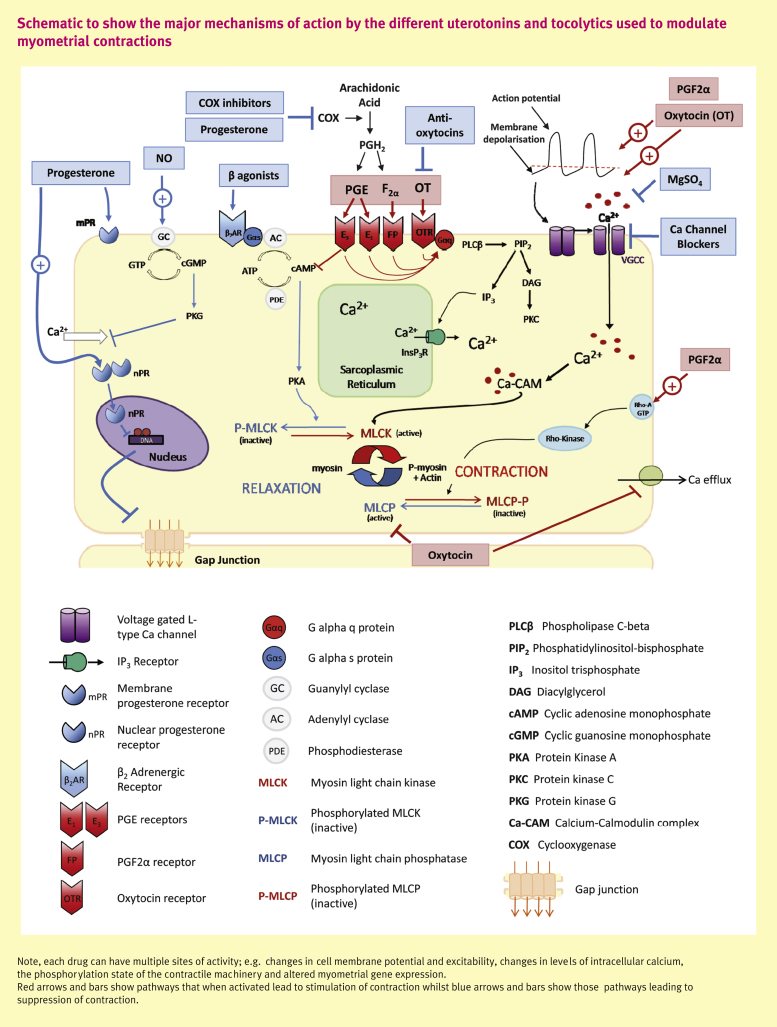
Schematic to show the major mechanisms of action by the different uterotonins and tocolytics used to modulate myometrial contractions

Phasic contractions of the uterus occur due to spontaneous changes in electrical activity within myometrial cells, the mechanism of which is unknown, but which leads to action potentials and depolarization. The depolarization causes opening of voltage-gated, L-type calcium channels, rapid calcium entry and an elevation in intracellular [Ca] ([Ca]*_i_*) which is pivotal for myometrial contraction. The Ca binds to calmodulin which activates myosin light chain kinase (MLCK), leading to phosphorylation of the myosin regulatory light chains and, enables the interaction of myosin with actin, cross-bridge cycling and force development. Myosin is dephosphorylated by myosin phosphatase (MLCP) and Ca is extruded by Ca-ATPase and Na/Ca exchange. The membrane potential is restored to resting levels by potassium (K) efflux and the myometrium is quiescent until spontaneous depolarization initiates the next contraction (see Figure 1, black arrows). Calcium entry and efflux are the major pathways of myometrial contraction/relaxation. Agonists by binding to their receptors on the myocyte membrane augment or inhibit this pathway, for example by promoting depolarization or by reducing Ca entry. However, agonists can also initiate other intracellular pathways and signals that can influence force. These primarily involve Ca uptake or release from the intracellular Ca store, the sarcoplasmic reticulum (SR) or modulation of MLCK/MLCP activity to change myosin and actin interaction (Ca sensitization). Control of these pathways is complex and incompletely understood, especially under physiological conditions; for example, second messengers have several sites of action and their effects may be antagonistic e.g. if PKC phosphorylates MLCK its activity is decreased and force falls, but when it phosphorylates MLCP, it decreases its activity hence prompting force.

In summary it is perhaps not surprising that the drugs (both clinical and laboratory) we have to modulate uterine force, act to alter the excitation–contraction that generates the phasic contractions of the uterus, and onto which agonists exert their effects.

## The uterotonins

### Oxytocin

**What is it:** oxytocin is a nona-peptide hormone released in pulses from the posterior pituitary and is the basis of Syntocin and Syntocinon.

**What does it do:** it has a number of roles including stimulating contraction of myometrium and myo-epithelium of the mammary ducts, and influencing maternal behaviour.

**How does it act:** activation of oxytocin receptors (OTR), in common with many other agonists, causes the activation of phospholipase-C β (PLCβ), which hydrolyses phosphatidylinositol bisphosphate (PIP_2_) leading to the formation of two second messengers: IP_3_ and diacylglycerol (DAG). Both messengers are thought to be involved in mediating the many cellular responses to oxytocin. IP_3_ stimulates calcium release from the SR, whilst DAG, the main activator of Protein Kinase C (PKC) may or may not affect myometrial tension. Using a variety of experimental approaches, we and others have shown that stimulation of Ca entry is the most prominent effect of oxytocin. Oxytocin also inhibits Ca efflux mechanisms. Oxytocin may also inhibit MLCP, slowing relaxation and enhancing force. Thus via a variety of mechanisms oxytocin has powerful stimulatory effects which have long been used clinically to aid parturition. During pregnancy, OTRs increase in number which is thought in part to underlie the increased sensitivity of the myometrium to oxytocin at term when little change in oxytocin levels can be detected. Antagonists to OTR have been developed as tocolytics and are discussed later.

### Clinical uses

The first clinical use of oxytocin was by Blair Bell in 1909, to stop post-partum haemorrhage. Oxytocin is now used widely in its synthetic forms, for labour augmentation and induction. Syntocin/Syntocinon is also administered during CS to cause a large contraction for stemming bleeding. Clinically, continuous iv infusion of oxytocin may not be optimal, as it does not replicate its natural pulsatile release and may also cause receptor desensitization and down regulation of OTR mRNA. Indeed, responses to administration of oxytocin are variable, and a pulsatile application of oxytocin has been shown to be more efficient than constant oxytocin infusion to induce labour. Even so it remains perplexing why up to 50% of women labouring poorly, do not respond to oxytocin administration and ultimately require CS, although difference in background acidity and lactate have recently been suggested. Thus dysfunctional labour remains a major contributor to the non-elective CS rate and oxytocin has not reduced this rate. More work is required to understand the causes of dysfunctional labour, so that they can either be prevented or remedied by additional agents.

Administration of oxytocin is not without risk; uterine hyperstimulation or rupture and foetal distress, The foetal distress arises as the over-contracted myometrium occludes blood vessels and diminishes placental perfusion. Thus the effects of administration of oxytocin in cases of poor/slow progress in the first stage of labour should always be carefully monitored and maximal doses, which are usually higher in primigravidae, not exceeded. In order to achieve successful labour induction with oxytocin, the cervix must be favourable.

### Prostaglandins

**What are they:** prostaglandins (PGs) are bioactive lipids derived from arachidonic acid. They are synthesized within the human foetal membranes (amnion and chorion) and decidua.

**What do they do:** they play a major role in both the initiation and maintenance of labour at term and also in some preterm labours, by enhancing contractions and “ripening” the cervix.

**How do they act:** they act as signalling messengers for many biochemical pathways. Their availability within the different tissues depends upon the activity of the different enzymes that converts their common precursor, arachidonic acid, into the various end products. PGs act in a paracrine or autocrine manner, mediating their functions by binding to different G protein coupled receptors (GPCRs). Each PG has its own specific receptor or receptors thus promoting the activation of different intracellular signalling cascades and gene transcription. When FP, EP1, EP3 and TP receptors are activated a contractile response occurs, whilst EP2, EP4, IP and DP receptors initiate relaxatory effects. In the myometrium, the two major stimulating PGs are PGF_2α_ and PGE. The levels of these two PGs are reported to increase in a time-dependent manner during later gestations indicating that they are important in the labour process. Both influence myometrial contractility but their effects vary (see Figure 1).

### PGF_2α_

PGF_2α_ exerts its uterotonic effect by increasing [Ca]*_i_* allowing for greater actin–myosin interaction and contraction, via (i) IP_3_-mediated SR calcium release, (ii) increasing Ca entry as a consequence of increasing the frequency of action potentials, and (iii) activation of non-specific cation channels, facilitating Ca entry. In human myometrium PGF_2α_ may also increase the sensitivity of the contractile apparatus to calcium. Thus as with oxytocin, multiple targets are activated by PGF_2α_ which synergizes to promote force production.

### PGE

There is controversy whether PGs of the ‘E’ variety aid myometrial contraction or relaxation. There are four isoforms of the PGE receptor (EP1–4), which act through different intracellular pathways: EP1 receptors couple to calcium mobilization and thus are uterotonins, EP3 receptors inhibit adenylyl cyclase and thus cAMP and Protein Kinase A. As both PKA and cAMP mostly relax the myometrium through a variety of mechanisms including decreasing Ca channel opening, phosphorylation of MLCK and stimulation of MLCP, EP_3_ will therefore be stimulatory. Conversely, EP2 and EP4 stimulate cAMP thereby mediating relaxation.

### Clinical uses

Clinically PGs have been used for many years for termination, and labour induction and for cervical preparation prior to the induction of labour. As natural PGs are rapidly metabolized, but labour induction requires their prolonged presence, PG analogues have been developed, e.g. Misoprostol and Gemeprost; synthetic analogues of PGE, which are used for medical termination. PGs are more efficacious for early terminations than oxytocin, as OTRs are not well expressed until the third trimester. The synthetic analogue of PGF2α, Carboprost (tradename Hemabate), has been used for stimulating labour contractions and reducing post-partum haemorrhage. Misoprostol has been successfully used for cervical ripening, but has also shown to increase the risk of uterine hyperstimulation and is therefore not always the preferred agent for labour induction. Despite a greater cost, Dinoprostone (also known as Cervidil, Prepidil or Prostin E2) is frequently used for cervical ripening owing to its greater safety.

## Tocolytics

### Introduction

Preterm labour, which remains a considerable obstetrical challenge, once initiated is considered to progress by the same mechanisms occurring in term labour but it is triggered is too early. The trigger may be physiological, for example as occurs in multiple pregnancy, or pathological for example due to infection. The inhibition of uterine contractions is the basis of drugs used in tocolysis. That there is no consensus about which agent is the best tocolytic, and that the rate of preterm delivery has not declined, tells the story of frustration and dashed expectations. Long-term (>1 week) tocolysis is rarely achieved, but the task of maintaining the pregnancy for an additional 48 h, can be successful, allowing corticosteroid administration and if necessary, transfer to a specialist centre.

### Progesterone

**What is it:** progesterone, (P4; pregn-4-ene-3,20-dione) is a steroid hormone. During pregnancy the main source of progesterone is placental tissues.

**What does it do:** progesterone is a ‘pro-gestational’ agent which maintains the pregnant state and promotes quiescence. Progesterone is effective in inhibiting contractions at all gestations. In other mammals, progesterone withdrawal initiates labour but no change occurs in serum levels in women, rather a process described as ‘functional progesterone withdrawal’ brought about by changes in receptor isoforms and reducing myometrial sensitivity to progesterone, is postulated.

**How does it act:** the primary action of progesterone is thought to be mediated by its interaction with the intracellular nuclear progesterone receptor however, more recently actions via a plasma membrane receptor (mPR) have been uncovered. It may also have anti-inflammatory actions which aids its tocolytic actions. Through its binding to nuclear receptors, progesterone alters gene expression bringing about long-term changes in the contractile phenotype of the myometrium. Progesterone inhibits phosphodiesterase PDE4, the enzyme responsible for cAMP inactivation, thereby increasing [cAMP]. Direct, rapid tocolytic effects of progesterone are associated with mPRs that couple to intracellular signalling pathways, although its exact mechanism of action is unclear. Progesterone *in vitro* inhibits spontaneous and oxytocin-induced contractions and uncouples the excitation–contraction process by directly modulating [Ca]*_i_* e.g. by inhibiting Ca entry and SR calcium release, as well as causing membrane hyperpolarization through activation of K channels. Progesterone may also inhibit oxytocin binding to its receptor. However, there are also reports of progesterone stimulating Ca entry and thus having some pre-contractile effects, at least acutely, and effects on K channels were not found in a recent study of human myometrium.

### Clinical uses

Progesterone is used to prevent preterm delivery. Supplementation with progesterone or the synthetic progestin, 17_α_-hydroxyprogesterone caproate (17OHPC) has been shown to be effective in preventing preterm labour in singleton deliveries but not multiple pregnancies. That said, there is little evidence of long-term benefit and The RCOG Preterm Birth study group has stated that progesterone use should be restricted to randomized controlled trials (see *Management of Preterm Labour* in this issue, pp 235–240).

When the effects of progesterone and its analogue were tested *in vitro* on myometrial strips, only progesterone and not 17OHPC was effective in reducing myometrial contractility, which is difficult to explain on our current understanding. Antiprogestins including Mifepristone (RU 486) have been developed to antagonize the action of progesterone and have clinical use for induction of labour or medical termination of pregnancy in early or mid trimester. Studies in mice have shown that Mifepristone can induce labour in late pregnancy whilst oral Mifepristone administration to women increased uterine activity.

### Magnesium

**What is it:** a co-factor for many reactions and an antagonist to Ca.

**What does it do:** it competes with Ca and thereby affects multiple intracellular pathways. It has long been used in obstetrics as a tocolytic and more recently to prevent and treat eclamptic convulsions.

**How does it act:** magnesium acts to relax smooth muscle and hence its administration is associated with vasorelaxation and tocolysis. Surprisingly, although MgSO_4_ was identified as a tocolytic over 40 years ago and is often the first line tocolytic in the USA, there is still uncertainty about the underlying mechanism. It acts extra- and intracellularly and decreases Ca entry and possibly SR Ca release. Physiologically there is an antagonism between Mg and Ca which reducing L-type Ca channel entry, one of its main actions. Magnesium induces a dose dependent decrease in [Ca]*_i_* and force in spontaneously and oxytocin-induced contracting pregnant human myometrium. Magnesium may decrease the generation of phospholipase-C and hence IP_3_ generation and stimulate translocation of PKC from cytosol to the cell membrane.

### Clinical uses

There is little evidence supporting MgSO_4_ efficacy in preventing preterm labour and delivery. Thus the 2009 Cochrane review concluded “Magnesium sulphate is ineffective at delaying birth or preventing preterm birth”, and of its use as a maintenance tocolytic concluded; “There is not enough evidence to show any difference between magnesium maintenance therapy and either placebo or no treatment”. This review follows several earlier studies reporting either no benefit or no difference from other tocolytics and even possible harm, although these conclusions are not supported by all. More recently, evidence has been presented suggesting that magnesium provides neuro-protection to the premature infant, possibly due to vasodilation and increased placental perfusion as well as NMDA antagonism. Finally, there appears to be little or no data demonstrating a prolongation of normal labour but an increase in CS rate. Magnesium deficiency may also contribute to pre-eclampsia and along with aspirin and Ca has been suggested as a supplement to prevent pre-eclampsia in high risk women.

### Calcium channel blockers

**What are they:** drugs such as nifedipine developed to block voltage-gated Ca channels were originally introduced to treat hypertension.

**What do they do:** prevent the normal rise of [Ca]*_i_* produced by stimulation and depolarization.

**How do they act:** blocking of calcium channels prevents the entry of Ca and the spread of the action potential, required for the myometrium to contract in a coordinated manner. *In vitro* nifedipine and other calcium channel blockers such as Diltiazem, reduce and then abolish the contractility of myometrium from preterm and term women, and this is associated with the abolition of Ca transients. The reduction in contractility has been reported to be less pronounced in samples from labouring women. Electrophysiological experiments on freshly isolated myocytes show nifedipine abolishes inward current. The uterus also expresses a small number of T-type Ca currents. Mibefradil, a blocker of T-type channels has been reported to cause a significant reduction in the contractility of pregnant human myometrium, but as a reduction was also found in calcium free medium, it is suggested to also inhibit calcium release from the SR.

### Clinical use

Compared to other tocolytic agents, calcium channel blockers significantly delay birth (7 days) when used before 34 weeks. Most of these trials involved the comparison of nifedipine with ritodrine, a beta adrenergic agent, discussed later. However, no placebo-control trials have addressed the acute management of preterm labour, and there is uncertainty about the optimal form, dose and route of administration for calcium channel blockers (see *Management of Preterm Labour* in this issue, pp 235–240). Nifedipine is associated with a reduction in the rate of respiratory distress syndrome, not just by allowing time for the use of corticosteroids but by a direct action on neonatal pulmonary perfusion. The side effect profile of calcium channel blockers has been heralded as superior to beta adrenergic agonists with fewer women stopping this treatment due to adverse effects and a reduction in neonatal side-effects such as respiratory distress syndrome, intraventricular haemorrhage and necrotizing enterocolitis. However, perhaps not surprisingly given its known use as an antihypertensive drug, cases of maternal hypotension and concerns about placental perfusion have been raised. It has been suggested that as calcium channel blockers are not licensed for tocolysis, under reporting of adverse events may hide the scale of this problem and caution is recommended, particularly in women with cardiovascular morbidity.

### Oxytocin antagonists as tocolytics

**What are they:** drugs which act as antagonists to the oxytocin receptor.

**What do they do:** they bind to OTR preventing the effects of oxytocin.

**How do they act:** the patho-physiology of preterm labour is still not understood however women with preterm labour were shown to have a higher sensitivity to oxytocin and a higher OTR concentration compared to women of a similar gestational age but not in labour. By preventing oxytocin binding this stimulation of uterine contractions should be inhibited. Atosiban, an oxytocin derivative is a competitive inhibitor of OTR as well as the vasopressin (V1a) receptor. *In vitro*, Atosiban was shown to abolish the uterotonic activity of oxytocin in human and animal myometrium and shown in parturient rats to down-regulate OXTRs. Barusiban, has a higher affinity for the OTR and higher potency than Atosiban, and is without side-effects of vasopressin receptor antagonism. *In vitro* it has been shown to inhibit contraction of human myometrium at least as potently as Atosiban.

### Clinical use

Atosiban was shown to achieve significant prolongation of gestation in a placebo-controlled trial. In the 2005 Cochrane intervention review of OTR antagonists for inhibiting preterm labour, Atosiban was superior neither to β_2_-AR agonists or placebo in both tocolysis and neonatal outcome. Furthermore, increased infant death was found. In a recent randomized controlled trial examining the effect of Barusiban on delivery and uterine contractility in women with threatened preterm labour at late gestational age, Barusiban was shown to be ineffective in delaying delivery or reducing uterine contractions. Thus it would seem that the early hope for anti-oxytocics has not been fulfilled.

### β-adrenergic receptor agonists

**What are they:** drugs developed to stimulate β_2_-adrenergic receptor (β_2_-AR), such as Terbutaline, Ritodrine, Salbutamol.

**What do they do:** relax smooth muscle including the myometrium.

**How do they act:** as agonists at β_2_-AR they activate adenylyl cyclase, thereby increasing cAMP and activating PKA. There are three subtypes of β-AR in the myometrium but β_2_-AR is the predominant (80%) form, although changes in receptor expression towards term and differences between women may occur. It has been postulated that a reduction or down regulation of β_2_-AR may have a role in the onset of preterm labour, although whether β_2_-AR decreases preceded the onset of labour or occurred as a consequence of labour is unclear. In rat myometrium progesterone but not oestradiol is associated with increased β_2_-AR transcription and elevation of β_2_-AR density. Moreover, progesterone has been shown to enhance the relaxant effect of ritodrine in gravid human myometrial samples, however, this may be due to direct inhibition of OTR and not the action of progesterone on the β_2_-AR, and has not been found by others. As well as the long-established ability of β_2_-AR agonists to relax the uterus, more recently several β_3_-AR agonists have been reported to reduce contractility of isolated myometrial strips in a dose dependent manner mediated via cAMP. Studies suggest that this effect is also mediated through an increase in cAMP. A study using implanted intrauterine telemetric sensors into pregnant monkeys demonstrated the *in vivo* tocolytic effect of a β_3_ agonist (SAR150640) and reduced side-effects compared to ritodrine. The β_3_-ARs also appear not to desensitize, a major drawback to β_2_-AR therapy. Further work is required to elucidate the role of β_3_ in the treatment of preterm labour.

### Clinical use

There is a large body of evidence supporting the use of β_2_-adrenergic receptor (β_2_-AR) agonists for the treatment of preterm labour, as they decrease the number of women delivering within 48 h. The extent of the tocolysis produced by β_2_ agonists is however limited. The widespread distribution of β_2_-AR results in an extensive sided effects profile, including palpitations, chest pain, shortness of breath, nausea, and in extreme cases pulmonary oedema and even maternal death, result in discontinuation of therapy. Desensitization also occurs and β_2_ agonists are not effective for maintenance therapy after preterm delivery has been abated. Unfortunately the delay in delivery produced by β_2_-AR agonists has not translated into an improvement in foetal outcome, although it must be noted that the use of corticosteroids for lung maturation was not routine when many of the trials were conducted. Terbutaline is also widely used clinically for hyperstimulation during labour. As β_2_-AR agonists occupy the receptor for a considerable length of time, they are irreversible in the short term, unless a β-blocking drug is given. Thus there is a risk of post-partum haemorrhage following β_2_-AR agonist tocolysis, as oxytocin is ineffective.

### Prostaglandin synthesis inhibitors

**What are they:** drugs that prevent the synthesis of prostaglandins. They include inhibitors of prostaglandin endoperoxide H synthase (PGHS), also known as cyclooxygenase (COX), familiar as non-steroidal antiflammatory drugs (NSAID) such as Indomethacin.

**What do they do:** decrease myometrial prostaglandin output.

**How do they act:** they inhibit the enzymes and pathways of prostaglandin production and thereby reduce myometrial contractions. There are two COX isoforms, COX1 and COX2 which bring about the conversion of arachidonic acid to PGH_2_ and hence the many different and tissue specific PGs. The COX1 enzyme is considered to be constitutively active and COX2 induced. The NSAIDs inhibit COX1 and COX2 and were first used as tocolytics, followed by the development of selective inhibitors for both COX1 and COX2, e.g. SC58560 (COX1) and celecoxib and meloxicam (COX2). *In vitro* data have demonstrated the inhibition of uterine contractions with NSAIDs, but not inhibition of PG, and a primary effect of these drugs on Ca channels has been postulated. In their study carefully examining a range of COX1 and COX2 inhibitors, Sawdy et al concluded that, as there was no relationship between myometrial inhibition and PG production, myometrial contractions are not dependent on myometrial COX activity or PG production, although *in vivo* local (e.g. foetal membrane) PG production may affect the myometrium. Another tocolytic approach is inhibition of PG receptors; THG113 a blocker of PGF receptors has been shown in animal models to delay premature delivery and reduce uterine activity.

### Clinical use

Several trials of COX inhibitors as tocolytics particularly indomethacin, have been undertaken. A meta-analysis of outcome data from 13 trials concluded “There is insufficient information on which to base decision about the role of COX inhibition for women in preterm labour”. Furthermore several serious side-effects have been reported, including necrotizing enterocolitis, patent postnatal ductus arteriosus and oligohydramnios which occurred when COX2 specific inhibitors were trialled. In the TOCOX trial of Rofecoxib, a newer COX2 inhibitor, no reduction in the incidence of preterm delivery occurred but it was associated with increased risk of delivery before 37 weeks in high risk women.

### Nitric oxide donors

**What are they:** producers of the short lived, metabolically active gas, NO e.g. glyceryl trinitrate.

**What do they do:** relax smooth muscle, particularly that of blood vessels.

**How do they act:** NO increases levels of cGMP and PKG and can thereby affect several pathways associated with relaxation. NO is formed from of L-arginine by the action of NO synthetase. Given its clear role in vasodilation via the endothelium it is not surprising that in reproduction a role for it has been suggested during implantation and menstruation and disorders of its production associated with pre-eclampsia, IUGR, dysmenorrhoea and infertility. There was considerable interest in NO as a tocolytic following firstly reports of its ability to inhibit *in vitro* uterine contractions and then, that the NO donor glyceryl trinitrate when used as a patch on the abdomen, could prevent preterm labour. However, since these early reports, contradictory results regarding the relaxant effects of NO appeared. One important issue has always been that there was much earlier evidence showing that cGMP does not relax the uterus, suggesting that NO must be effecting another pathway. Buxton et al provide evidence that this may be a K channel-mediated mechanism.

### Clinical use

Nitric oxide donors have been employed for cervical ripening, labour induction and tocolysis. In a 2002 intervention review, five randomized controlled trials were identified (466 women) and nitroglycerine was the NO donor in all five studies. The main finding was that NO did not delay labour or improve neonatal outcome compared to placebo or alternative tocolytic, although it was well tolerated. In 2004 the RNOTT trial of NO tocolysis reported that NO (GTN) is less efficacious than β_2_-AR agonists.

## Summary

An understanding of the mechanism of excitation–contraction in myometrium provides a good basis for predicting tocolytic targets. When translating this knowledge into producing therapeutic agents several concerns are raised which focus around specificity and efficacy. Thus for example, it is hard to suggest that MgSO_4_ or nifedipine will produce selective effects on myometrium given the widespread distribution of L-type Ca channels and the multitude of reactions including Mg. Desensitization and side-effects are other problems encountered that limit long-term use. Comparison of trial outcomes between existing tocolytics or placebo can be found in sources such as Cochrane Reviews, and often these make depressing reading in terms of proven tocolytic efficacy e.g. for Mg, or in effectiveness of new drugs, such as oxytocin receptor antagonists. It is worth noting that one of the oldest tocolytics around, progesterone is currently enjoying a renaissance, at least for singleton pregnancies. The goal of preventing preterm births is such a worthy one that research into both the underlying causes and devising better drugs must continue. We also suggest that focus on the drugs that meta-analysis has shown to be superior e.g. prostaglandin inhibitors and calcium channel blockers.

**Practice points**

• The excitation–contraction pathway is the target for all uterotonic and tocolytics drugs, thus an understanding of it is helpful.
• Ultimately a rise of intracellular [Ca] determines uterine contraction and a fall of [Ca] relaxation.
• Oxytocin and prostaglandin and their synthetic derivatives are the most commonly used uterine stimulants. They are used to speed up labour, stem bleeding, termination and labour induction but have the risk of uterine hyperstimulation and rupture.
• Once preterm labour is initiated there is a very powerful driving force for contraction which is hard to halt and reverse. Tocolysis beyond 48 h remains a significant challenge, but progesterone and Ca channel blockers can be used.
• There is a lack of supporting evidence for the use of some tocolytics and some may do harm. Thus well designed trials and new approaches to tocolysis are required.
